# Mitigate SIR epidemic spreading via contact blocking in temporal networks

**DOI:** 10.1007/s41109-021-00436-w

**Published:** 2022-01-06

**Authors:** Shilun Zhang, Xunyi Zhao, Huijuan Wang

**Affiliations:** Faculty of Electrical Engineering, Mathematics, and Computer Science, Delft University of Technology, Mekelweg 4, 2628 CD Delft, The Netherlands

**Keywords:** Epidemic mitigation, Temporal network, Contact blocking

## Abstract

Progress has been made in how to suppress epidemic spreading on temporal networks via blocking all contacts of targeted nodes or node pairs. In this work, we develop contact blocking strategies that remove a fraction of contacts from a temporal (time evolving) human contact network to mitigate the spread of a Susceptible-Infected-Recovered epidemic. We define the probability that a contact *c*(*i*, *j*, *t*) is removed as a function of a given centrality metric of the corresponding link *l*(*i*, *j*) in the aggregated network and the time *t* of the contact. The aggregated network captures the number of contacts between each node pair. A set of 12 link centrality metrics have been proposed and each centrality metric leads to a unique contact removal strategy. These strategies together with a baseline strategy (random removal) are evaluated in empirical contact networks via the average prevalence, the peak prevalence and the time to reach the peak prevalence. We find that the epidemic spreading can be mitigated the best when contacts between node pairs that have fewer contacts and early contacts are more likely to be removed. A strategy tends to perform better when the average number contacts removed from each node pair varies less. The aggregated pruned network resulted from the best contact removal strategy tends to have a large largest eigenvalue, a large modularity and probably a small largest connected component size.

## Introduction

Networks, such as physical contact networks and online social networks, facilitate the spread of epidemics and information. The study of epidemic spreading first assumed the topology of networks to be static (Pastor-Satorras et al. 2015; Wang et al. 2013), while many real-world networks are not static as nodes and links can appear and disappear over time, thus can be better represented as temporal networks (Holme and Saramäki 2012). For example, human contact networks such as face-to-face contact networks (Zhao et al. 2011) are temporal networks, which can be described by a sequence of contacts (or temporal links) between pairs of individuals occurring at discrete time steps. The increasing availability of network data with temporal information has fostered research on how the temporal aspect of networks can affect dynamic processes such as the spreading of epidemics (Zhang et al. 2017; Karsai et al. 2011) and information (Scholtes et al. 2014) on temporal networks. Epidemic/information spreading can be mitigated via reducing physical contacts. Covid-19 measures like curfew, working at home, social distancing all aim to block physical contacts. These measures treat at least a subgroup of the population in the same way. In this work, we address the further question of how to mitigate the epidemic spreading more effectively via selecting the contacts to block heterogeneously and strategically. We propose to develop contact removal strategies utilizing the network properties of contacts.

We consider real-world physical contact networks, where only the connection between nodes evolves (appears when there is a contact and disappears) over time whereas the nature/type of nodes and contacts do not change . In this case, a temporal network observed within a time window [0, *T*] can be represented by $\mathcal {G}=(\mathcal {N},\mathcal {C})$, where $\mathcal {N}$ is the node set observed within [0, *T*], size $N=|\mathcal {N}|$ is the number of nodes in the network, $\mathcal {C}=\{c(i,j,t), t\in [0,T],i,j\in \mathcal {N}\}$ is the set of contacts between pairs of nodes in $\mathcal {N}$, with contact (*i*, *j*, *t*) representing the interaction between node *i* and node *j* at time step *t*. A contact *c*(*i*, *j*, *t*), also called a temporal link, describes interaction/connection between node *i* and *j* at a specific time *t*. A node without any contact at time *t* can be regarded as inactive or not observed at that time step. We confine ourselves to the Susceptible-Infected-Recovered (SIR) epidemic spreading model (Pastor-Satorras et al. 2015) on a temporal network instead of more realistic spreading processes: Initially at $t=0$, a seed node is selected to be infected whereas all the other nodes are susceptible; When a contact happens between an infected node and a susceptible node at any time step, the susceptible node becomes infected with a probability $\beta$; Each infected node becomes recovered with a probability $\gamma$ at each time step. A recovered node will neither be infected nor infect any other node. The contacts to block will be selected based on the (time) aggregated network $\mathcal {G_W}$ of the temporal network $\mathcal {G}$. Aggregated network represented as $\mathcal {G_W}=(\mathcal {N}, \mathcal {L})$ is a weighted network with the same node set $\mathcal {N}$ as temporal network $\mathcal {G}$, $\mathcal {L}$ is the set of weighted links, two nodes *i* and *j* in $\mathcal {G_W}$ are connected by a link *l*(*i*, *j*) if they have at least one contact in temporal network $\mathcal {G}$ and link *l*(*i*, *j*) is associated with a weight recording the number of contacts in $\mathcal {G}$ between the two nodes. In the rest of this paper, links refer to the links in the aggregated network, and contacts will not be called temporal links anymore to avoid confusion. Contacts between two nodes *i* and *j* can be regarded as the corresponding link *l*(*i*, *j*) in the aggregated network activated at specific time steps.

The objective is to mitigate the epidemic spreading via blocking a given percentage $\phi$ of contacts, selected based on the aggregated network. The fraction $\phi$ of contacts removed corresponds to the cost of the mitigation. To launch a contact removal intervention during the time window [0, *T*], the information of the aggregated network of the temporal network $\mathcal {G}$ observed in [0, *T*] needs to be known at $T=0$. Such aggregated network is assumed to be given in our work, whereas in practice, it can be estimated based on the temporal network observed before $T=0$. Predicting the aggregated network is more feasible compared to predicting the temporal network in [0, *T*]. The latter, i.e. long-term prediction of time specific and possibly noisy contacts challenges machine learning approaches that target at short-term predictions. Hence, we focus on the development of contact removal strategies based on the aggregated network, instead of the complete temporal network information which is difficult to obtain.

We propose probabilistic contact removal strategies. Specifically, the probability that a contact *c*(*i*, *j*, *t*) is removed is a generic function of a centrality metric (Newman 2018) of link *l*(*i*, *j*) in the aggregated network and the time *t* of the contact. Each centrality metric leads to a unique mitigation strategy in contact removal. The impact of an SIR epidemic spreading can be evaluated via the following performance measures, which will be used to evaluate the mitigation strategies: the average prevalence over time, where the prevalence at a time step is the number of infected nodes; the maximal prevalence, so called peak height, which suggests the maximal demand in e.g. hospital resources; the time to reach the peak prevalence, so called peak time, which indicates the time to prepare the medical resources for the peak demand.

The mitigation strategies that we have proposed are evaluated in 6 real-world temporal networks. We find that the mitigation effect is better when contacts between node pairs that have fewer contacts are removed with a higher probability. Removing contacts that occur earlier in time could further enhance the mitigation effect. A strategy tends to better mitigate the epidemic spreading if the average number of contacts removed varies less among node pairs. Furthermore, we analyze properties of the aggregated pruned network resulted from each contact blocking strategy. We find that the optimal strategy tends to lead to an aggregated pruned network with a large largest eigenvalue, a large modularity and a possibly a small largest connected component. Networks with a large modularity and a small largest connected component are difficult for an epidemic to spread. Static networks with a small largest eigenvalue have been shown to be robust against epidemic spreading i.e. have a high epidemic threshold for Susceptible-Infected-Susceptible epidemic. The resultant aggregated pruned network after contact removal, however, may lead to a low prevalence if its largest eigenvalue is large. This suggests that the temporal information of contacts, may lead to new phenomena that can not be captured by static network studied.

Recent work has been devoted to understand the influence of temporal networks on dynamic processes and especially the mitigation of epidemic spreading. A first line of reseach has studied the mitigation of epidemic spreading via node-level approaches. Génois et al. (2015) have shown that vaccination of individuals who act as bridges between communities in time-aggregated network can efficiently prevent epidemic outbreaks. Gemmetto et al. (2014) have investigated the epidemic mitigation via excluding a sub-group of nodes in a temporal network in school environments. Another line of research has focused on link-based approaches to suppress epidemic outbreaks. Link removal strategies based on link centrality metrics in the aggregated network has been studied in Zhan et al. (2019). These strategies select the links in the aggregated network to block, thus removing all contacts associated with the selected links. In this work, we investigate in-depth at contact level, i.e. how to select a given number of contacts to remove to suppress epidemic spreading. To the best of our knowledge, few works have studied contact-level approaches to suppress epidemic spreading. Our previous work (Zhao and Wang 2020) has addressed the same question, however, was confined to Susceptible-Infected (SI) model, which is a special case of SIR model. In this work, we consider the SIR model, broaden and deepen our investigation towards a more comprehensive evaluation of mitigation effect and a more systematic analysis of the properties of the pruned network to explain the performance of the strategies. In view of the uncertainty of realistic temporal network data, we further check the robustness of our finding in the relative effectiveness of proposed mitigation strategies when the temporal networks are under the perturbation, i.e. when the time (ordering) of contacts is uncertain.

## Methods

We will firstly propose our contract removal strategies. Afterwards, we will introduce the real-world temporal networks and simulations that will be used to simulate the epidemic spreading process and further to evaluate the effect of the mitigation strategies.

### Contact blocking strategies

We select the contacts to block based on a given centrality metric in the aggregated network and the time of each contact. Specifically, the probability that a contact *c*(*i*, *j*, *t*) is removed is defined as a function of the given centrality metric of the corresponding link *l*(*i*, *j*) in the aggregated network $\mathcal {G_W}$ and the time *t* of the contact. This function also ensures that a fraction $\phi$ of contacts are removed on average.

#### Link centrality metrics

We propose a set of link centrality metrics based on node centrality metrics for the aggregated network $\mathcal {G_W}$. The aggregated network $\mathcal {G_W}$ is a weighted network constructed from a temporal network $\mathcal {G}$. The weight of each link in the aggregated network represents the number of contacts between the two corresponding nodes in the temporal network. Each centrality metric below will lead afterwards to a unique mitigation strategy:

- *Degree product* of a link *l*(*i*, *j*) refers to $d(i)\cdot d(j)$, where *d*(*i*) is the degree of node *i* defined as the number of links incident to node *i* in the aggregated network.
- *Strength product* of a link *l*(*i*, *j*) refers to $s(i)\cdot s(j)$, where *s*(*i*) is the strength of node *i* defined as the total weights of all the links incident to node *i* in aggregated network. The strength of a node tells the total number of contacts the node has.
- *Betweenness* is the number of shortest paths that traverse the link between all possibly node pairs in the unweighted aggregated network (Wang et al. 2008).
- *Link weight* of a link *l*(*i*, *j*) in aggregated network refers to the total number of contacts between node *i* and *j* in the corresponding temporal network.
- *Weighted eigenvector component product* is the product of the principal eigenvector components of the link’s two end nodes. The principal eigenvector is the eigenvector corresponds to the largest eigenvalue of the weighted aggregated network.
- *Unweighted eigenvector component product* is the product of the principal eigenvector components of the link’s two end nodes. The principal eigenvector is the eigenvector corresponds to the largest eigenvalue of the unweighted aggregated network.

Besides the proposed strategies based on the aforementioned link centrality metrics, we introduce a baseline strategy called *Random removal*. In the *Random removal* strategy, the probability for each contact *c*(*i*, *j*, *t*) to be removed is independent of the centrality of *l*(*i*, *j*). Or equivalently, *Random removal* sets the centrality value as 1 for all links.

#### Contact removal probability

Given a link centrality metric *m*, we can derive the centrality $m_{ij}$ for each link *l*(*i*, *j*) in the aggregated network. Consider the simple case where the probability that a contact *c*(*i*, *j*, *t*) between *i* and *j* is removed is independent of the time *t* and we first propose the removal preference $p_{ij}$:

1 $$\begin{aligned} p_{ij} = m_{ij}\frac{\phi \sum _{lk} w_{lk}}{\sum _{lk} (w_{lk}m_{lk})} \end{aligned}$$

where $w_{ij}$ is the weight of link *l*(*i*, *j*) in the aggregated network or equivalently the number of contacts between *i* and *j*, $\phi$ is the expected fraction of contacts to be removed, thus we have $\sum _{ij} p_{ij}w_{ij}=\phi \sum _{lk} w_{lk}$, which is the expected number of contacts to be removed. The removal preference $p_{ij}$ of a contact between any node pair *i* and *j* is proportional to the centrality $m_{ij}$ of the corresponding link *l*(*i*, *j*).

We cannot use the removal preference $p_{ij}$ directly as the removal probability of a contact between node *i* and *j* in view of the following. Some centrality metrics could be highly heterogeneous. The removal preference $p_{ij}$ is possibly larger than 1 if the centrality measure $m_{ij}$ of the link *l*(*i*, *j*) is large. To deal with this issue, we propose an iterative process to derive the contact removal probability by re-normalizing $p_{ij}$, where ${i},{j}\in \mathcal {N}$: we assign removal probabilities 1 to those contacts whose removal preference $p_{ij}$ according to (1) is larger than one, and re-normalize $p_{ij}$ among the contacts with $p_{ij}\le 1$ to satisfy $\sum _{ij} p_{ij}w_{ij}=\phi \sum _{ij} w_{ij}$. We repeat this normalization process until the removal preference $p_{ij}$ of all contacts are between 0 and 1, while the actual average fraction of contacts blocked is $\phi$. Now we define $\tilde{p}_{ij}$ as the re-normalized $p_{ij}$ via the proposed iterative process, and $\tilde{p}_{ij}$ is used as the removal probability of each contact between node *i* and node *j*.

We further generalize the definition of the contact removal preference $p_{ij}$ as

2 $$\begin{aligned} p^*_{ij} = m_{ij}^{\alpha }\frac{\phi \sum _{lk} w_{lk}}{\sum _{lk} (w_{lk}m_{lk}^{\alpha })} \end{aligned}$$

The removal preference of a contact *c*(*i*, *j*, *t*) is proportional to a polynomial function of $m_{ij}$. The definition (1) of $p_{ij}$ is a special case when $\alpha =1$ of definition (2). The random strategy, i.e. all contacts have the same probability of being removed, corresponds to the case when $\alpha =0$. Consider (1) where the reciprocal metric $\frac{1}{m_{ij}}$ is taken as a new centrality metric. The corresponding strategy is equivalent to the general definition (2) where metric $m_{ij}$ is considered and $\alpha =-1$.

In this work, we consider the definition (1) of $p_{ij}$ using the aforementioned list of centrality metrics and their reciprocals as well as the random strategy, which correspond to the general definition of (2) where $\alpha =1,-1,0$, respectively.

Finally, we generalize our strategy by considering the timestamps of the contacts. This is motivated by the intuition that early intervention, e.g. blocking early contacts, could be possibly more effective. We propose a time-dependent contract removal preference $p_{ij}(t)$:

3 $$\begin{aligned} p_{ij}(t) = m_{ij}f(t)\frac{\phi \sum _{lk} w_{lk}}{\sum _{lk} (w_{lk}m_{lk}f(t))} \end{aligned}$$

where *f*(*t*) describes the preference to remove contacts at specific period. The preference that *c*(*i*, *j*, *t*) is removed is proportional to $m_{ij}\cdot f(t)$. The same aforementioned normalization process is applied to this generalized contact removal preference to derive the removal probability of each contact.

As a start, we consider $f(t)=4\cdot 1_{t\le T/2}+1_{t>T/2}$, $f(t)=1_{t\le T/2}+4 \cdot 1_{t>T/2}$ and $f(t)=1$, where the indicator function $1_{y}$ is one if the condition *y* is true, and otherwise it is 0. They correspond to the preference of removing contacts happening early in [1, *T*/2], late in (*T*/2, *T*] and no preference for the timestamps of the contacts, respectively.

### Datasets

The following real-world physical contact networks will be considered:

- HighSchool11&12 record the physical contacts between students in a high school in Marseilles, France (Fournet and Barrat 2014). The two datasets consider two different groups of students.
- WorkPlace13&15 capture the contacts between individuals in an office building in France (Génois et al. 2015). The two datasets are measured from different groups of individuals respectively.
- MIT are human contact network among students of the Massachusetts Institute of Technology (Kunegis 2013; Eagle and Pentland 2006). The MIT dataset has been measured for about 8 months.

All networks are undirected. Their properties are given in Table 1. The duration of each time step is either 1 s or 20 s in all the networks. For the MIT dataset, we choose randomly two observation period, each of about one-week time. The temporal networks corresponding to these two periods are called MIT1 and MIT2. In this way, all the six temporal networks (HighSchool11&12, WorkPlace13&15, MIT1&2) are comparable in observation window. They will be used to study the impact of the mitigation strategies on the average prevalence over time, the focus of this work.

**Table 1:** Basic properties of real-world networks: the number of nodes, links (in the aggregated network) and contacts, respectively

Datasets	Nodes	Links	Contacts	Duration
HighSchool11	126	1709	28561	3.15
HighSchool12	180	2220	45047	8.44
WorkPlace13	92	755	9827	11.43
WorkPlace15	217	4274	78249	11.50
MIT1	74	355	29107	6.99
MIT2	45	200	22714	6.99
MIT	96	5078	1086404	232.30

The duration refers to the duration *T* of the observation window [1,T] in the units of days

However, most networks have a short duration of the observation window, within 12 days, besides MIT. In order to observe the peak (increase and afterwards decrease of) prevalence in the SIR process, the observation window of a temporal network needs to be long in duration. When we study the performance measure like peak height/prevalence and peak time, we repeat each of the temporal network HighSchool11&12, WorkPlace13&15 respectively for 10 times. The constructed networks, *HighSchool11&12, *WorkPlace13&15 which repeats one temporal network periodically are also called periodic networks (Zhang et al. 2017). Each constructed network has a duration ten times as large as the original network . We consider the 4 constructed network *HighSchool11&12, *WorkPlace13&15 and the MIT dataset to study the performance of the strategies in terms of peak prevalence and peak time.

### Simulation

In this subsection, we will introduce the simulation of the SIR spreading process and the choice of parameters. The performance measures to evaluate the mitigation strategies will be discussed in the next section.

We consider the following discrete time SIR spreading process: a seed node is chosen to be infected at $t=0$, while the other nodes are susceptible at $t=0$. Each contact between an infected node and a susceptible node could lead to an infection with probability $\beta$. At each time step, each infected node recovers with a recovery probability $\gamma$. We consider infection probability $\beta =0.01$ as an example. In this case and when $\gamma =0$, the prevalence at *T* is around the order of $10\%$ in the first six temporal networks. Furthermore, we consider the recover probability per time step $\gamma = 1.22*10^{-6}$ or $\gamma = 0$. The former, $\gamma = 1.22*10^{-6}$ leads approximately to a recovery probability $10\%$ per day.

In the simulation, we simulate the exact infection and recovery process except the following approximation in the recovery process. If there is no contact in the whole network for the period $t_0,t_0+t$, we update the state of each node only at the end of this time window $t_0+t$ instead of at each of the *t* time steps. In the datasets we have considered, the longest gap that no contact happens is around one day. Correspondingly, the average prevalence is the number of infected nodes over the time steps when at least one contact happens in the network.

Given a temporal network and a centrality metric, we compute the contact removal preference (1) for each contact based on the aggregated network of the temporal network and derive further the removal probability of each contact via the normalization process of the contact removal preference. We select each node as a possible seed node and iterate the following for five times per seed node: the fraction $\phi$ of contacts to be removed are selected according to contact removal probabilities; The SIR process starting from the given seed is performed on the pruned temporal network resulted from the removal of the selected contacts; the prevalence $\rho$ is recorded at each time step when there is a contact in the network. Given a network and a centrality measure, we obtain the prevalence at a time step as the average over the five iterations per every seed node. The average prevalence over all time steps when there is at least one contact is used as the key performance to evaluate the contact removal strategies. The fraction $\phi$ of contacts to be removed is a control parameter and $\phi =10\%$ and $\phi =30\%$ are considered. Simulations are performed in the same way when the time factor *f*(*t*) are taken into account via the contact removal probability (3).

## Results

In this section, we evaluate our contact removal strategies via three performance measures: the average prevalence and the peak height (the maximal number of infected at a time step) and the peak time (the time to reach the peak height/prevalence.

### Performance evaluation

#### Average prevalence

**Fig. 1:**
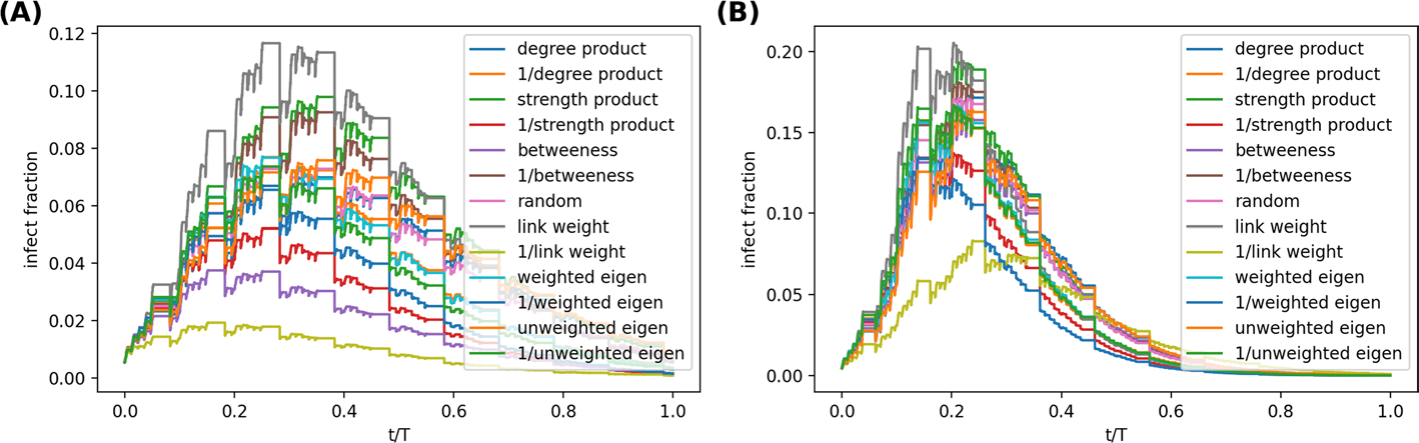
The prevalence $\rho$ of the SIR model over time in periodic network *HighSchool12 (**A**) and *WorkPlace15 (**B**), when mitigated via 13 contact blocking strategies defined by (1) respectively. The infection rate is $\beta =0.01$ per time step, the recovery rate is $\gamma = 1.22*10^{-6}$ per time step, approximately $10\%$ per day and $30\%$ of the contacts are removed

Firstly, we evaluate the strategies as defined in (1) where the probability that a contact *c*(*i*, *j*, *t*) is removed is independent of the time *t* of the contact but do depend a centrality metric of the link *l*(*i*, *j*) in the aggregated network. In total, 13 strategies are considered that correspond to the aforementioned centrality metrics and their reciprocals. Figure 1 exemplifies the prevalence $\rho (t)$ over time in two periodic networks *HighSchool12 and *WorkPlace15 when each of the 13 strategies is performed and $10\%$ contacts are removed. The ordering of the prevalence $\rho (t)$ at each time step for the 13 strategies are relatively stable over time. The relative performance of the mitigation strategies in terms of average prevalence over time seems not sensitive to duration of the observation time window.

We use the original network HighSchool11&12, WorkPlace13&15, MIT1&2 to evaluate the blocking strategies with respect to the average prevalence. These networks are comparable in duration of the observation time window, i.e. within 12 days. The performance of each strategy in each network is evaluated via the the average prevalence $E[\rho ]$, i.e., the average fraction of infected nodes over the time steps when there is at least one contact in the network.

We start with the simple case when the recovery rate $\gamma =0$. In this case, the SIR model is equal to the Susceptible-Infected (SI) model. The average prevalence when contacts are removed according to each strategy are shown in Tables 2 and 3, where $\phi =10\%$ and $\phi =30\%$ contacts are removed respectively. In most networks, the 1/link weight performs the best among all 13 strategies. The same has been observed when the recovery rate is $\gamma = 1.22*10^{-6}$ per step, approximately $10\%$ per day, as shown in Tables 4 and 5. These observations suggest that removing contacts between nodes that have few contacts tends to be the most effective in reducing the average prevalence.

**Table 2:** The average prevalence $E[\rho ]$ when the recovery rate is $\gamma = 0\%$ per step, and $\phi =10\%$ of the contacts are removed from each temporal network using removal probability (1) based on each centrality metric

Metrics	HighSchool11	HighSchool12	WorkPlace13	WorkPlace15	MIT1	MIT2
degree product	0.043	0.038	0.027	0.102	0.106	0.193
1/degree product	0.044	0.041	0.028	0.107	0.097	0.183
strength product	0.049	0.042	0.028	0.106	0.110	0.193
1/strength product	0.046	0.040	0.027	0.108	0.098	0.164
betweeness	0.046	0.037	0.027	0.106	0.097	0.178
1/betweeness	0.047	0.041	0.028	0.109	0.112	0.189
random	0.045	0.040	0.028	0.106	0.109	0.202
link weight	0.052	0.042	0.028	0.122	0.111	0.189
1/link weight	**0.038**	**0.032**	**0.025**	**0.084**	**0.084**	0.159
weighted eigen	0.050	0.041	0.028	0.108	0.121	0.197
1/weighted eigen	0.048	0.040	0.027	0.107	0.095	**0.158**
unweighted eigen	0.041	0.040	0.027	0.100	0.104	0.196
1/unweighted eigen	0.046	0.040	0.029	0.107	0.099	0.187

The lowest average prevalence value for each dataset is highlighted in bold

**Table 3:** The average prevalence $E[\rho ]$ when the recovery rate is $0\%$ per step, and $\phi =30\%$ of the contacts are removed from each temporal network using removal probability (1) based on each centrality metric

Metrics	HighSchool11	HighSchool12	WorkPlace13	WorkPlace15	MIT1	MIT2
degree product	0.026	0.026	0.021	0.057	0.084	0.184
1/degree product	0.037	0.031	0.024	0.072	0.073	0.142
strength product	0.038	0.029	0.024	0.068	0.099	0.184
1/strength product	0.030	0.028	0.022	0.063	0.063	0.109
betweeness	0.032	0.026	0.022	0.059	0.074	0.151
1/betweeness	0.032	0.030	0.023	0.068	0.102	0.164
random	0.032	0.027	0.022	0.064	0.088	0.168
link weight	0.043	0.034	0.024	0.088	0.107	0.183
1/link weight	**0.020**	**0.018**	**0.020**	**0.038**	**0.055**	0.119
weighted eigen	0.032	0.031	0.023	0.070	0.101	0.177
1/weighted eigen	0.043	0.030	0.024	0.070	0.064	**0.099**
unweighted eigen	0.026	0.027	0.022	0.056	0.092	0.167
1/unweighted eigen	0.040	0.030	0.023	0.075	0.080	0.141

﻿The lowest average prevalence value for each dataset is highlighted in bold

**Table 4:** The average prevalence $E[\rho ]$ when the recovery rate is $\gamma = 1.22*10^{-6}$ per step, approximately $10\%$ per day, and $\phi =10\%$ of the contacts are removed from each temporal network using removal probability (1) based on each centrality metric

Metrics	HighSchool11	HighSchool12	WorkPlace13	WorkPlace15	MIT1	MIT2
degree product	0.034	0.023	0.014	0.051	0.074	0.132
1/degree product	0.038	0.023	0.014	0.052	0.071	0.124
strength product	0.038	0.024	0.014	0.051	0.069	0.131
1/strength product	0.037	0.023	0.013	0.049	0.061	**0.110**
betweeness	0.037	0.024	0.013	0.050	0.064	0.130
1/betweeness	0.038	0.023	0.015	0.050	0.072	0.130
random	0.036	0.024	0.014	0.047	0.075	0.126
link weight	0.043	0.024	0.015	0.058	0.078	0.139
1/link weight	**0.031**	**0.020**	**0.013**	**0.040**	**0.061**	0.111
weighted eigen	0.038	0.024	0.014	0.051	0.078	0.133
1/weighted eigen	0.039	0.024	0.014	0.055	0.072	0.122
unweighted eigen	0.033	0.022	0.013	0.045	0.076	0.138
1/unweighted eigen	0.039	0.024	0.013	0.050	0.068	0.127

The lowest average prevalence value for each dataset is highlighted in bold

**Table 5:** The average prevalence $E[\rho ]$ when the recovery rate is $\gamma = 1.22*10^{-6}$ per step, approximately $10\%$ per day, and $\phi =30\%$ of the contacts are removed from each temporal network using removal probability (1) based on each centrality metric

Metrics	HighSchool11	HighSchool12	WorkPlace13	WorkPlace15	MIT1	MIT2
degree product	0.021	0.015	0.011	0.026	0.060	0.118
1/degree product	0.032	0.018	0.012	0.033	0.051	0.093
strength product	0.031	0.017	0.011	0.031	0.069	0.121
1/strength product	0.024	0.016	0.011	0.030	0.044	0.075
betweeness	0.025	0.015	0.012	0.027	0.050	0.108
1/betweeness	0.026	0.018	0.011	0.030	0.070	0.110
random	0.026	0.016	0.012	0.031	0.062	0.105
link weight	0.036	0.021	0.012	0.040	0.068	0.121
1/link weight	**0.017**	**0.011**	**0.010**	**0.017**	**0.039**	0.081
weighted eigen	0.027	0.018	0.012	0.032	0.062	0.124
1/weighted eigen	0.037	0.018	0.012	0.034	0.042	**0.068**
unweighted eigen	0.021	0.016	0.011	0.026	0.063	0.119
1/unweighted eigen	0.032	0.018	0.012	0.034	0.056	0.094

The lowest average prevalence value for each dataset is highlighted in bold

Furthermore, we consider the time dependent contact removal strategies where the contact removal probability $p_{ij}(t)$ is defined in (3). When $f(t)=4\cdot 1_{t\le T/2}+1_{t>T/2}$, a contact happening early in time i.e. $t<T/2$ is 4 times more likely to be removed than a contact occurring late $t>T/2$. When $f(t)=1_{t\le T/2}+4\cdot 1_{t>T/2}$, contacts happening late i.e. $t>T/2$ are more likely to be removed. Contact removal strategies based on each of these two *f*(*t*) examples and each centrality metric are evaluated via the average prevalence. Their performance when $\gamma =0$, $\phi =10\%$ is shown in Tables 6 and 7, where early and later contacts are more likely removed respectively. Comparing these results and the time-independent strategies (Table 3) or equivalently when $f(t)=1$, we find that removing earlier contacts better suppresses the epidemic spreading. The same has been observed when $\gamma =1.22*10^{-6}$, $\phi =10\%$ (see Tables 8 and 9). Moreover, metric 1/link weight tends to have the best performance independent of the choice of *f*(*t*). Therefore, the epidemic spreading can be better mitigated when contacts between node pairs that have few contacts and happening early are more probable to be removed.

**Table 6:** The average prevalence $E[\rho ]$ when the recovery rate is $\gamma = 0\%$ per step and $\phi =10\%$ of the contacts are removed from each temporal network using removal probability (3) based on each centrality metric and $f(t)=4\cdot 1_{t\le T/2}+ 1_{t>T/2}$. Contacts occurring early in time i.e. $t<T/2$ are more likely to be removed

Metrics	HighSchool11	HighSchool12	WorkPlace13	WorkPlace15	MIT1	MIT2
degree product	0.040	0.037	0.027	0.101	0.109	0.191
1/degree product	0.044	0.038	0.028	0.106	0.097	0.171
strength product	0.045	0.039	0.027	0.109	0.107	0.184
1/strength product	0.044	0.039	0.026	0.100	0.091	0.159
betweeness	0.041	0.034	0.027	0.098	0.099	0.165
1/betweeness	0.044	0.040	0.027	0.102	0.107	0.185
random	0.041	0.037	0.028	0.101	0.102	0.184
link weight	0.049	0.040	0.028	0.122	0.118	0.188
1/link weight	**0.035**	**0.030**	**0.026**	**0.080**	**0.081**	0.160
weighted eigen	0.045	0.040	0.028	0.108	0.096	0.192
1/weighted eigen	0.047	0.041	0.028	0.102	0.098	**0.159**
unweighted eigen	0.038	0.038	0.027	0.097	0.104	0.197
1/unweighted eigen	0.050	0.041	0.029	0.107	0.103	0.170

The lowest average prevalence value for each dataset is highlighted in bold

**Table 7:** The average prevalence $E[\rho ]$ when the recovery rate is $\gamma = 0\%$ per step and $\phi =10\%$ of the contacts are removed from each temporal network using removal probability (3) based on each centrality metric and $f(t)=1_{t\le T/2}+4\cdot 1_{t>T/2}$. Contacts occurring late in time i.e. $t>T/2$ are more likely to be removed

Metrics	HighSchool11	HighSchool12	WorkPlace13	WorkPlace15	MIT1	MIT2
degree product	0.043	0.040	0.027	0.106	0.109	0.193
1/degree product	0.047	0.042	0.028	0.110	0.102	0.186
strength product	0.051	0.042	0.027	0.109	0.111	0.200
1/strength product	0.045	0.040	0.028	0.105	0.095	0.172
betweeness	0.046	0.038	0.027	0.107	0.101	0.191
1/betweeness	0.046	0.042	0.027	0.111	0.115	0.193
random	0.048	0.041	0.028	0.107	0.108	0.200
link weight	0.051	0.045	0.029	0.114	0.114	0.191
1/link weight	**0.041**	**0.035**	**0.026**	**0.086**	**0.089**	**0.161**
weighted eigen	0.048	0.041	0.028	0.112	0.108	0.191
1/weighted eigen	0.050	0.041	0.028	0.112	0.097	0.166
unweighted eigen	0.046	0.040	0.027	0.107	0.109	0.200
1/unweighted eigen	0.050	0.043	0.027	0.108	0.103	0.191

The lowest average prevalence value for each dataset is highlighted in bold

**Table 8:** The average prevalence $E[\rho ]$ when the recovery rate is $\gamma = 1.22*10^{-6}$ per step, and $\phi =10\%$ of the contacts are removed from each temporal network using removal probability (3) based on each centrality metric and $f(t)=4\cdot 1_{t\le T/2}+ 1_{t>T/2}$. Contacts occurring early in time i.e. $t<T/2$ are more likely to be removed

Metrics	HighSchool11	HighSchool12	WorkPlace13	WorkPlace15	MIT1	MIT2
degree product	0.032	0.023	0.013	0.046	0.070	0.127
1/degree product	0.039	0.025	0.014	0.049	0.065	0.113
strength product	0.038	0.024	0.014	0.051	0.072	0.133
1/strength product	0.037	0.023	0.013	0.048	0.062	0.108
betweeness	0.034	0.021	0.014	0.047	0.062	0.114
1/betweeness	0.033	0.023	0.014	0.048	0.069	0.134
random	0.033	0.023	0.013	0.048	0.070	0.131
link weight	0.037	0.024	0.013	0.054	0.079	0.131
1/link weight	**0.029**	**0.019**	**0.012**	**0.038**	**0.054**	**0.104**
weighted eigen	0.036	0.025	0.013	0.050	0.067	0.128
1/weighted eigen	0.038	0.025	0.014	0.050	0.064	0.111
unweighted eigen	0.030	0.023	0.013	0.044	0.073	0.126
1/unweighted eigen	0.038	0.024	0.014	0.051	0.068	0.113

The lowest average prevalence value for each dataset is highlighted in bold

**Table 9:** The average prevalence $E[\rho ]$ when the recovery rate is $\gamma = 1.22*10^{-6}$ per step and $\phi =10\%$ of the contacts are removed from each temporal network using removal probability (3) based on each centrality metric and $f(t)=1_{t\le T/2}+4\cdot 1_{t>T/2}$. Contacts occurring late in time i.e. $t>T/2$ are more likely to be removed

Metrics	HighSchool11	HighSchool12	WorkPlace13	WorkPlace15	MIT1	MIT2
degree product	0.036	0.025	0.014	0.052	0.074	0.134
1/degree product	0.039	0.024	0.014	0.053	0.071	0.124
strength product	0.039	0.025	0.014	0.052	0.078	0.139
1/strength product	0.037	0.024	0.014	0.052	0.070	0.117
betweeness	0.037	0.024	0.014	0.051	0.065	0.126
1/betweeness	0.036	0.025	0.014	0.051	0.077	0.133
random	0.037	0.025	0.014	0.051	0.077	0.138
link weight	0.041	0.026	0.015	0.055	0.075	0.129
1/link weight	**0.033**	**0.021**	0.014	**0.042**	**0.059**	**0.110**
weighted eigen	0.040	0.025	0.014	0.051	0.082	0.141
1/weighted eigen	0.041	0.025	0.014	0.054	0.067	0.115
unweighted eigen	0.036	0.025	**0.013**	0.050	0.078	0.136
1/unweighted eigen	0.039	0.024	0.014	0.056	0.067	0.123

The lowest average prevalence value for each dataset is highlighted in bold

#### Properties of the pruned network

The pruned network is the resultant temporal network after contacts being removed according to a strategy. In this section, we explore the relation between the properties of the pruned network and the average prevalence, resulted from a contact removal strategy. This could help us understand what kind of pruned networks may lead to a low prevalence. We focus on time-independent contact removal strategies to illustrate our method.

**Fig. 2:**
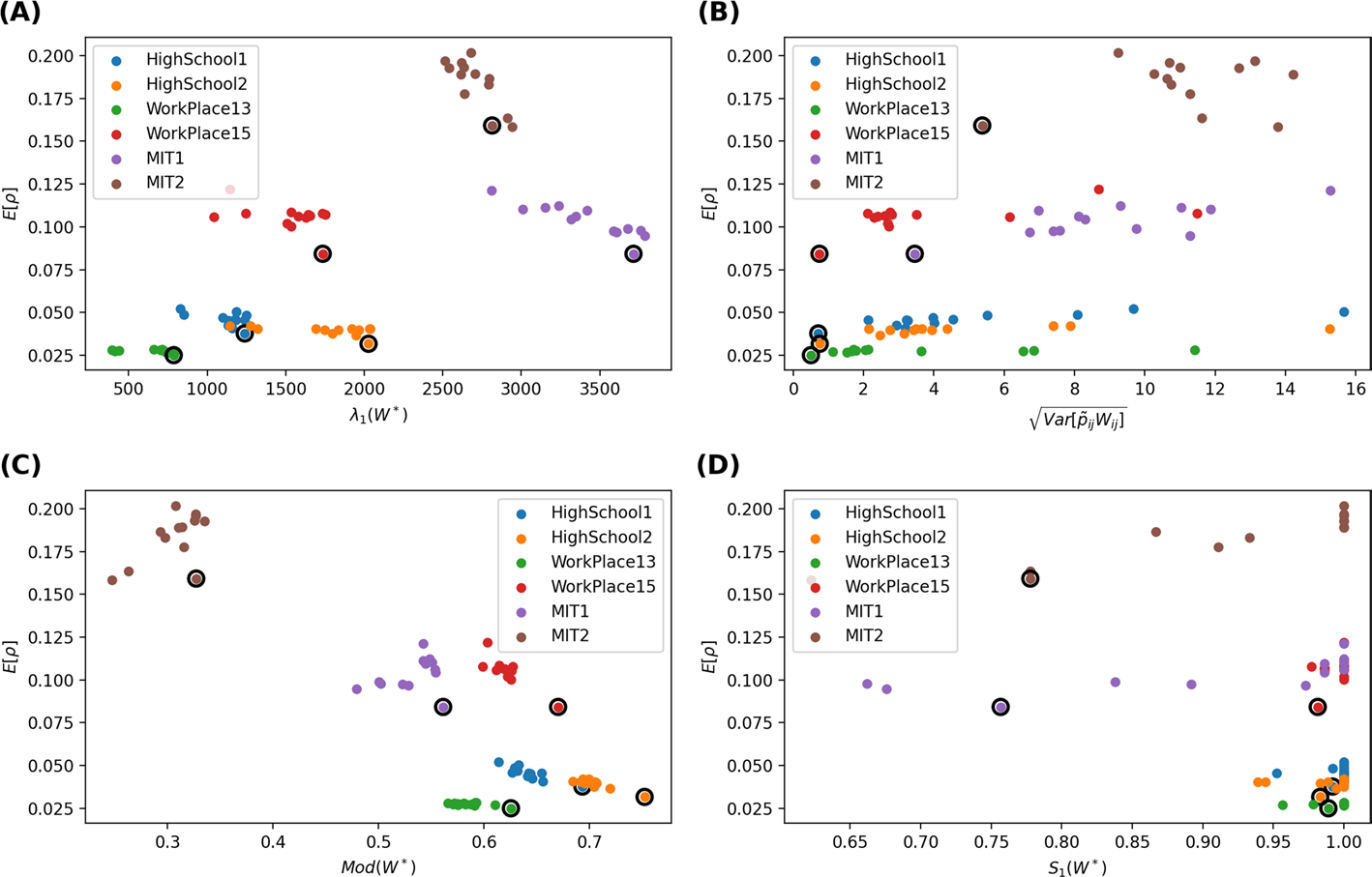
Scatter plot of the average prevalence $E[\rho ]$ versus the largest eigenvalue $\lambda _1(W^*)$ of the aggregated pruned network (**A**), the standard deviation $\sqrt{Var[\tilde{p}_{ij}w_{ij}]}$ of the average number of contacts removed from a node pair (**B**) the modularity $Mod(W^*)$ (**C**) and the relative size of the largest connected component of the aggregated pruned network (**D**), respectively. A fraction $\phi =10\%$ of the contacts are removed. The recovery rate is $\gamma = 0$ per step. The results obtained with 1/link weight strategy are circled

**Fig. 3:**
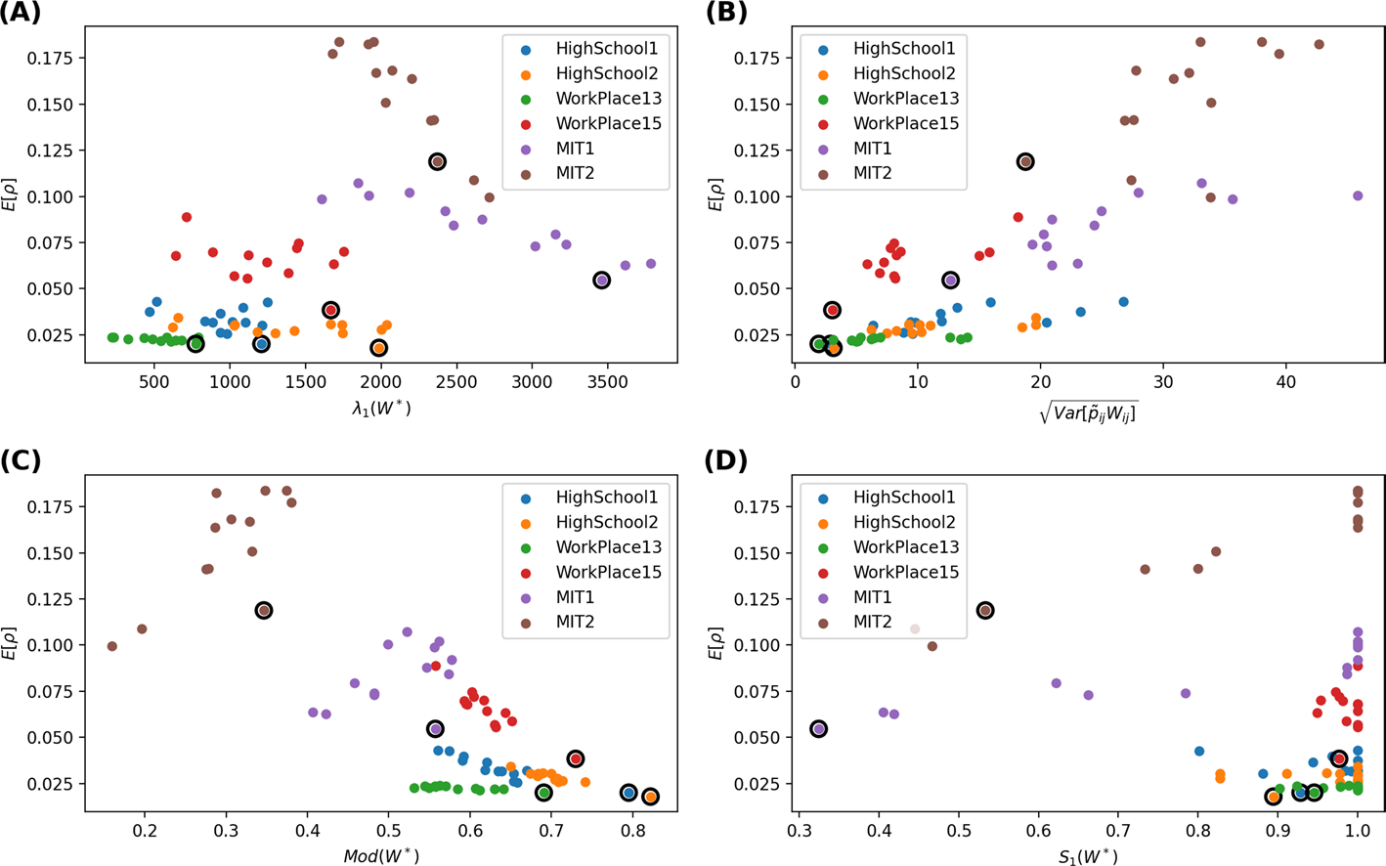
Scatter plot of the average prevalence $E[\rho ]$ versus the largest eigenvalue $\lambda _1(W^*)$ of the aggregated pruned network (**A**), the standard deviation $\sqrt{Var[\tilde{p}_{ij}w_{ij}]}$ of the average number of contacts removed from a node pair (**B**) the modularity $Mod(W^*)$ (**C**) and the relative size of the largest connected component of the aggregated pruned network (**D**), respectively. A fraction $\phi =30\%$ of the contacts are removed. The recovery rate is $\gamma = 0$ per step. The results obtained with 1/link weight strategy are circled

**Fig. 4:**
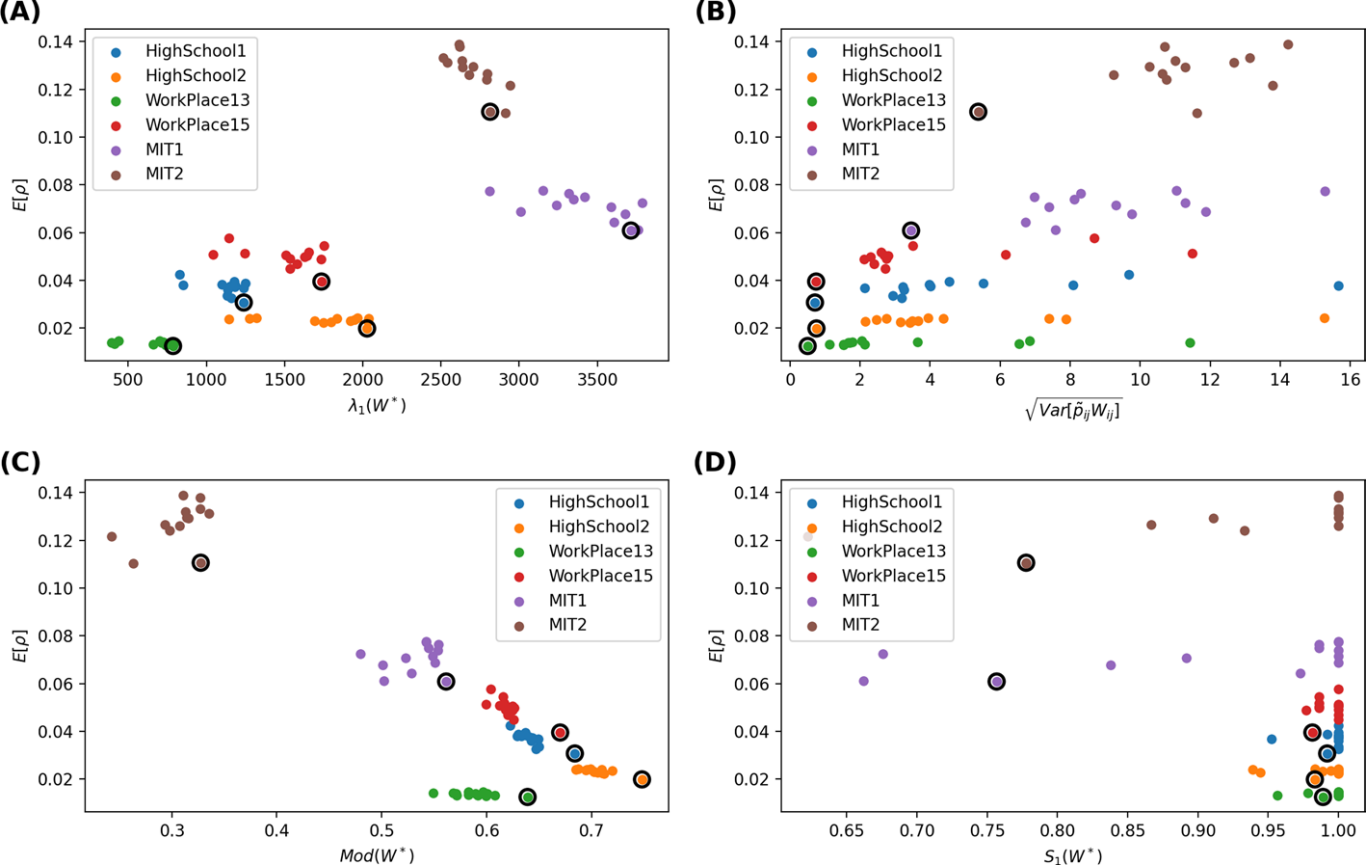
Scatter plot of the average prevalence $E[\rho ]$ versus the largest eigenvalue $\lambda _1(W^*)$ of the aggregated pruned network (**A**), the standard deviation $\sqrt{Var[\tilde{p}_{ij}w_{ij}]}$ of the average number of contacts removed from a node pair (**B**) the modularity $Mod(W^*)$ (**C**) and the relative size of the largest connected component of the aggregated pruned network (**D**), respectively. A fraction $\phi =10\%$ of the contacts are removed. The recovery rate is $\gamma = 1.22*10^{-6}$ per step, approximately $10\%$ per day. The results obtained with 1/link weight strategy are circled

**Fig. 5:**
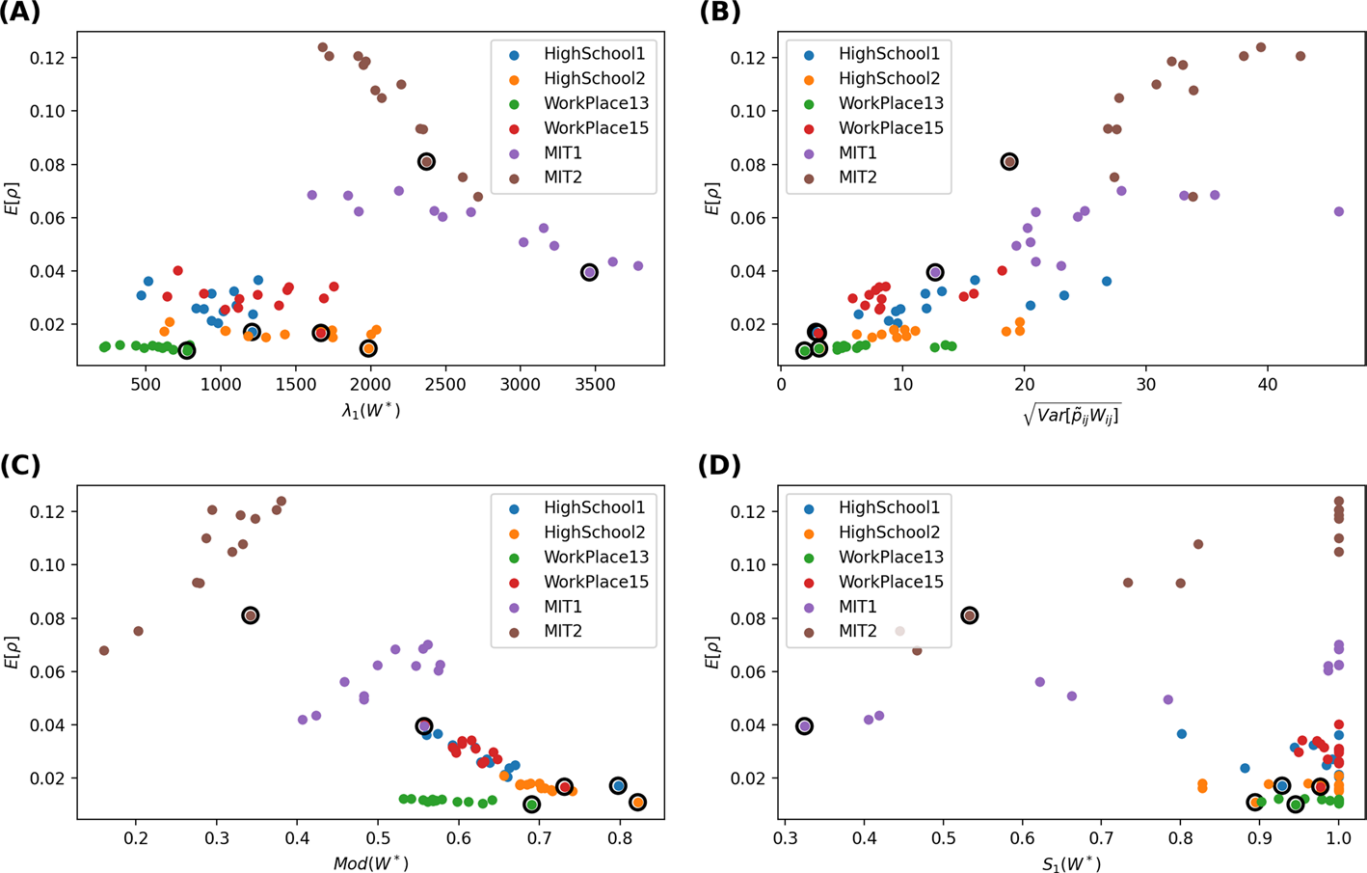
Scatter plot of the average prevalence $E[\rho ]$ versus the largest eigenvalue $\lambda _1(W^*)$ of the aggregated pruned network (**A**), the standard deviation $\sqrt{Var[\tilde{p}_{ij}w_{ij}]}$ of the average number of contacts removed from a node pair (**B**) the modularity $Mod(W^*)$ (**C**) and the relative size of the largest connected component of the aggregated pruned network (**D**), respectively. A fraction $\phi =30\%$ of the contacts are removed. The recovery rate is $\gamma = 1.22*10^{-6}$ per step, approximately $10\%$ per day. The results obtained with 1/link weight strategy are circled

The average number of contacts removed between any node pair *i* and *j* or link *l*(*i*, *j*) in the aggregated network is ${\tilde{p}}_{ij}w_{ij}$, where $w_{ij}$ is the number of contacts between *i* and *j* and ${\tilde{p}}_{ij}$ is the probability that a contact between *i* and *j* is removed. The average number of contacts removed by strategy 1/link weight is the same for all links in the aggregated network^1^. We explore whether a strategy that removes a similar number of contacts per node pair (link) may better mitigates the epidemic spreading. Figure 2b demonstrates the scatter plot of the average prevalence $E[\rho ]$ versus $\sqrt{Var[\tilde{p}_{ij}w_{ij}]}$ for each strategy when $\phi =10\%$ contacts are removed and the recovery rate is $\gamma = 0$ per step. We find that, in each network, a strategy tends to reduce the average prevalence $E[\rho ]$ more if $\sqrt{Var[\tilde{p}_{ij}w_{ij}]}$ is small. The same can be observed when the recovery rate $\gamma$ and removal fraction $\phi$ vary (see (b) of Figs. 3, 4, 5).

Each pruned network is a temporal network. We investigate three properties of the aggregated network $W^{*}$ of the pruned network. Each element $W^{*}_{ij}$ in the weighted adjacency matrix $W^{*}$ of the aggregated pruned network tells the number of contacts between *i* and *j* in the pruned network.

We explore firstly the largest eigenvalue $\lambda _{1}(W^*)$ of the aggregated pruned network in relation the corresponding average prevalence resulted from each strategy. Consider the Susceptible-Infected-Susceptible SIS epidemic spreading process on a static network. It has been shown that the largest eigenvalue of the network suggests the robustness of the network subject to epidemic spreading (Van Mieghem et al. 2008; Wang et al. 2013; Ottaviano et al. 2019; Qu and Wang 2017). When the effective infection rate, i.e. infection rate divided by the recovery rate, is above (below) the threshold $\tau _{c}\sim \frac{1}{\lambda _{1}(W^*)}$, a none-zero (zero) fraction of the population is infected in the meta-stable state. A static network whose largest eigenvalue is small has a large epidemic threshold, thus is robust against epidemic spreading.

Would a pruned network with a small $\lambda _{1}(W^*)$ lead to a low prevalence according to the findings of SIS model on static networks? Figures 2a, 3a, 4a, 5a respectively show the scatter plot of the average prevalence $E[\rho ]$ versus $\lambda _{1}(W^*)$ of the aggregated pruned network^2^ for each strategy in each network. We observe the opposite: the best strategy with the lowest prevalence tends to lead to a pruned network with a large largest eigenvalue. Such inconsistency can be possibly explained as follows. First, a network that is robust against SIS epidemic spreading is not necessarily robust against SIR epidemic spreading. Each link in the aggregated pruned network can transmit the epidemic maximally once in SIR model whereas possibly multiple times in SIS models. That is why removing many contacts from links whose end nodes have a high strength may better reduce the largest eigenvalue and better suppress the SIS epidemic but not the SIR epidemic spreading. Second, a network with a low epidemic threshold does not implies a high prevalence when the effective infection rate is above the epidemic threshold. Finally, the aggregated pruned network can not capture the temporal information of contacts, which influence the spread of an epidemic.

Furthermore, we consider the modularity $Mod(W^*)$ of the aggregated pruned network. Given a weighted network and a given partition of all the nodes into non-overlapping communities, the quality of this community partition can be measured by the modularity (Newman 2006; Ge and Wang 2012) $\frac{1}{2L}\sum _{i,j=1}^{N}{(W^*_{ij}-\frac{s_{i}s_{j}}{2L})\delta _{C_{i}C_{j}}}$, where $s_{i}$ is the strength of node *i*, $C_{i}$ is the label of the community to which node *i* belongs to, the Kronecker delta function $\delta _{C_{i}C_{j}}=1$ if $C_{i}=C_{j}$ or else $\delta _{C_{i}C_{j}}=0$. The modularity of a partition describes the extent to which that more link weights are within each community than link weights between communities. The modularity $Mod(W^*)\in [0,1]$ of a network is the maximal modularity that could be obtained via network/node partition. We compute the modularity of an aggregated pruned network via the Louvain method (Blondel et al. 2008). The scatter plot in Figs 2c, 3c, 4c, 5c shows that the optimal contact removal strategy that obtains the minimal average prevalence tends to result in a pruned network that has a large modularity. A network with a large modularity is more robust against epidemic spreading.

Finally, we explore the relative size $S_{1}(W^*)$ of the largest connected component of the aggregated pruned network. We wonder whether the optimal strategy reduced the prevalence via disconnecting the network. As shown in the bottom-right figure of Figs. 2d, 3d, 4d, 5d, most pruned networks still have a relative large component $S_{1}(W^*) \sim 1$. Exceptions are observed for in MIT1 and MIT2, where strategies may evidently disconnect the aggregated pruned network. In such cases, the optimal strategy tends to lead to a relatively small largest component size $S_{1}(W^*)$. This is in line with the finding that efficient immunization strategy should keep the largest connected component size small (Schneider et al. 2011).

In summary, the optimal mitigation strategy tends to lead to an aggregated pruned network with a large largest eigenvalue, a large modularity and possibly a small largest connected component (in case contact removal strategies evidently disconnect the pruned network). Moreover, a strategy seems to better reduce the prevalence if it removes a similar number of contacts from the links. These observations together further support our previous explanation why the optimal strategy could result in an aggregated pruned network with a large largest eigenvalue: the optimal strategy tends to remove a similar number of contacts from links, keeping the hubs, i.e. nodes with a large node strength. Such hubs contribute to a large largest eigenvalue and thus a low epidemic threshold for SIS epidemic spreading. However, the modular structure of the pruned network limits the prevalence of an epidemic, which can not be captured directly by the largest eigenvalue.

#### Peak height and peak time

The peak height/prevalence and peak time suggest the maximal demand in e.g., health-care resources and the time to prepare for the highest demand in resources, respectively. We consider the 4 constructed network *HighSchool11&12, *WorkPlace13&15 and the MIT dataset to study the performance of the strategies in terms of peak prevalence and peak time.

For each centrality metric or strategy, we simulate the SIR spreading process five times for every possible seed node. The peak height is found as the maximum prevalence in each spreading process. Table 10 shows the average peak height over all 5*N realizations of the spreading processes. We find that the strategy 1/link weight results in the smallest peak height. The average peak height shown in Table 10 differs from the maximal prevalence in Fig. 1, which corresponds to the maximum of the average prevalence over the 5*N realizations.

**Table 10:** The peak height i.e. the highest prevalence over time, when the recovery rate is $10\%$ per day, and $\phi =10\%$ of the contacts are removed from each temporal network using removal probability (1)

Metrics	HighSchool11	HighSchool12	WorkPlace13	WorkPlace15	MIT
degree product	0.355	0.121	0.018	0.235	0.163
1/degree product	0.346	0.119	0.020	0.239	0.156
strength product	0.356	0.130	0.020	0.246	0.182
1/strength product	0.301	0.109	0.017	0.225	0.130
betweeness	0.314	0.099	0.018	0.230	0.171
1/betweeness	0.354	0.120	0.020	0.242	0.164
random	0.346	0.119	0.018	0.236	0.167
link weight	0.384	0.132	0.020	0.261	0.170
1/link weight	**0.279**	**0.077**	**0.016**	**0.193**	**0.110**
weighted eigen	0.371	0.128	0.020	0.237	0.182
1/weighted eigen	0.298	0.115	0.021	0.227	0.144
unweighted eigen	0.347	0.117	0.019	0.235	0.169
1/unweighted eigen	0.343	0.112	0.018	0.241	0.164

The lowest peak height for each dataset is highlighted in bold

Similarly, the average peak time, i.e. time to reach the maximal prevalence over all spreading processes started at every possible seed node is derived and given in Table 11. Interestingly, the peak time for strategy 1/link weight is not always the smallest. Strategy 1/link weight leads to the lowest peak height and possibly a longer peak time.

**Table 11:** The peak time in units of *t*/*T* before the maximum prevalence is achieved. The recovery rate is $10\%$ per day, and $\phi =10\%$ of the contacts are removed from each temporal network using removal probability (1)

Metrics	HighSchool11	HighSchool12	WorkPlace13	WorkPlace15	MIT
degree product	3.714	2.518	0.877	1.381	0.429
1/degree product	3.514	2.519	1.695	1.380	0.429
strength product	3.317	2.520	1.678	1.381	0.429
1/strength product	3.321	2.522	1.258	1.382	0.430
betweeness	3.717	2.778	1.208	1.381	0.429
1/betweeness	3.320	2.521	1.078	**1.380**	0.429
random	3.120	2.521	1.145	1.381	0.429
link weight	3.119	**2.516**	1.297	1.381	**0.429**
1/link weight	4.119	2.719	**0.617**	1.904	0.589
weighted eigen	3.120	2.519	1.111	1.381	0.430
1/weighted eigen	**3.117**	2.519	1.314	1.381	0.429
unweighted eigen	3.915	2.520	1.079	1.381	0.482
1/unweighted eigen	3.120	2.519	1.043	1.381	0.482

The shortest peak time for each dataset is highlighted in bold

#### Robustness

Temporal networks measured in real-world scenarios possibly contain noise, e.g., uncertainty of the ordering of contacts or occurring time of contacts. We would like to explore whether our findings in the relative effectiveness of the proposed mitigation strategies still holds when the temporal networks measured are subject to such type of uncertainty.

We assume the temporal networks that we have so far analyzed are measured relatively precisely. For each of these temporal networks, we apply two approaches, respectively, to generate the corresponding temporal networks perturbed by the aforementioned uncertainty. The duration of one time step in the original temporal networks is either 1 s or 20 s. We split the observation period [0, *T*] of a temporal network into non-overlapping bins, whose duration is $\Delta =60$ s to further perturb the networks. We first adopt the uncertainty model I used in Antulov-Fantulin et al. (2015), which randomly reshuffles the timestamps of the contacts within each bin of $\Delta =60$ s. This model encapsulates the uncertainty of the ordering of contacts that happen at similar time. Given the uncertainty model I (one network realization as an example) of each original temporal network, we evaluate the contact blocking strategies in the same way as in the original network and their performance is given in Tables 12 and 13. We find that the ranking of the strategies does not change in model I compared to that in the original temporal networks and the 1/link weight remains the best strategy. Our finding seems to be robust against minor uncertainty in the ordering of contacts.

**Table 12:** The average prevalence $E[\rho ]$ in uncertainty model I when the recovery rate is $\gamma = 0$ per step, and $\phi =30\%$ of the contacts are removed from each temporal network using removal probability (1) based on each centrality metric

Metrics	HighSchool11	HighSchool12	WorkPlace13	WorkPlace15	MIT1	MIT2
degree product	0.028	0.027	0.022	0.059	0.089	0.180
1/degree product	0.038	0.031	0.025	0.072	0.075	0.142
strength product	0.038	0.029	0.023	0.069	0.101	0.186
1/strength product	0.030	0.027	0.022	0.065	0.061	0.107
betweeness	0.031	0.025	0.022	0.061	0.073	0.154
1/betweeness	0.036	0.030	0.023	0.064	0.100	0.175
random	0.033	0.027	0.023	0.063	0.092	0.165
link weight	0.042	0.033	0.024	0.087	0.105	0.185
1/link weight	**0.021**	**0.018**	**0.020**	**0.037**	**0.056**	0.124
weighted eigen	0.034	0.032	0.023	0.068	0.104	0.187
1/weighted eigen	0.047	0.030	0.024	0.071	0.060	**0.101**
unweighted eigen	0.026	0.028	0.022	0.053	0.097	0.176
1/unweighted eigen	0.041	0.030	0.024	0.074	0.077	0.142

The lowest average prevalence value for each dataset is highlighted in bold

**Table 13:** The average prevalence $E[\rho ]$ in uncertainty model I when the recovery rate is $\gamma = 1.22*10^{-6}$ per step, approximately $10\%$ per day, and $\phi =30\%$ of the contacts are removed from each temporal network using removal probability (1) based on each centrality metric

Metrics	HighSchool11	HighSchool12	WorkPlace13	WorkPlace15	MIT1	MIT2
degree product	0.022	0.016	0.013	0.026	0.060	0.112
1/degree product	0.031	0.018	0.012	0.033	0.052	0.098
strength product	0.030	0.018	0.012	0.030	0.068	0.120
1/strength product	0.025	0.017	0.012	0.031	0.042	0.081
betweeness	0.024	0.016	0.011	0.027	0.048	0.095
1/betweeness	0.027	0.018	0.012	0.031	0.068	0.115
random	0.026	0.017	0.011	0.030	0.055	0.112
link weight	0.036	0.021	0.013	0.041	0.064	0.125
1/link weight	**0.017**	**0.011**	**0.010**	**0.017**	**0.035**	0.082
weighted eigen	0.027	0.019	0.012	0.033	0.066	0.120
1/weighted eigen	0.035	0.019	0.013	0.032	0.042	**0.067**
unweighted eigen	0.021	0.017	0.011	0.025	0.065	0.121
1/unweighted eigen	0.033	0.018	0.012	0.034	0.051	0.096

The lowest average prevalence value for each dataset is highlighted in bold

To capture the uncertainty of the exact occurring time of contacts, we use our uncertainty model II, where each contact’s occurring time is measured in the time resolution of $\Delta =60$ s instead of second. In other words, the number of contacts between each pair of nodes in each bin of $\Delta =60$ s is known in model II. However, the exact occurring time of the contacts happening within each bin in precision of seconds is unknown. For each snapshot/bin of $\Delta =60$ s, model II constructs a weighted network, where the weight between two nodes counts the number of contacts between them that occur within the bin of $\Delta =60$ s. Each weighted network is thus an aggregated network of the original temporal network over 60 s. The performance of each blocking strategy on model II are shown in Tables 14 and 15, demonstrating that strategy 1/link weight outperforms the others, the same as observed in the original temporal networks. Hence, our evaluation of the strategies is robust against network perturbations that models the uncertainty of temporal network data.

**Table 14:** The average prevalence $E[\rho ]$ in uncertainty model II when the recovery rate is $\gamma = 0$ per step, and $\phi =30\%$ of the contacts are removed from each temporal network using removal probability (1) based on each centrality metric

Metrics	HighSchool11	HighSchool12	WorkPlace13	WorkPlace15	MIT1	MIT2
degree product	0.022	0.016	0.011	0.025	0.061	0.114
1/degree product	0.031	0.018	0.013	0.033	0.047	0.093
strength product	0.029	0.017	0.012	0.031	0.065	0.122
1/strength product	0.026	0.017	0.012	0.032	0.041	0.077
betweeness	0.025	0.015	0.012	0.029	0.051	0.103
1/betweeness	0.027	0.017	0.013	0.030	0.068	0.113
random	0.028	0.017	0.012	0.030	0.061	0.109
link weight	0.035	0.022	0.013	0.043	0.073	0.128
1/link weight	**0.017**	**0.011**	**0.011**	**0.018**	**0.038**	0.086
weighted eigen	0.028	0.019	0.012	0.033	0.070	0.130
1/weighted eigen	0.036	0.018	0.012	0.034	0.043	**0.068**
unweighted eigen	0.019	0.015	0.011	0.025	0.066	0.118
1/unweighted eigen	0.032	0.018	0.011	0.036	0.053	0.096

The lowest average prevalence value for each dataset is highlighted in bold

**Table 15:** The average prevalence $E[\rho ]$ in uncertainty model II when the recovery rate is $\gamma = 1.22*10^{-6}$ per step, and $\phi =30\%$ of the contacts are removed from each temporal network using removal probability (1) based on each centrality metric

Metrics	HighSchool11	HighSchool12	WorkPlace13	WorkPlace15	MIT1	MIT2
degree product	0.021	0.016	0.012	0.022	0.055	0.114
1/degree product	0.026	0.017	0.012	0.030	0.051	0.099
strength product	0.027	0.017	0.012	0.026	0.069	0.110
1/strength product	0.023	0.016	0.011	0.029	0.039	0.078
betweeness	0.023	0.015	0.012	0.025	0.051	0.095
1/betweeness	0.024	0.017	0.012	0.028	0.060	0.109
random	0.022	0.016	0.011	0.028	0.058	0.103
link weight	0.030	0.020	0.013	0.036	0.066	0.117
1/link weight	**0.016**	**0.011**	**0.011**	**0.017**	**0.036**	0.081
weighted eigen	0.025	0.017	0.013	0.029	0.060	0.123
1/weighted eigen	0.029	0.018	0.011	0.031	0.041	**0.066**
unweighted eigen	0.020	0.015	0.011	0.024	0.061	0.108
1/unweighted eigen	0.028	0.017	0.012	0.032	0.051	0.094

The lowest average prevalence value for each dataset is highlighted in bold

## Conclusions

In this paper, we have developed and evaluated contact blocking strategies in order to mitigate SIR epidemic spreading on a temporal network. The probability that a contact *c*(*i*, *j*, *t*) is removed is defined as a generic function of a given centrality metric of the corresponding link *l*(*i*, *j*) in the corresponding aggregated network and time *t*. In total 12 centrality metrics or strategies and a baseline strategy (random removal) have been considered. The strategy (1/link weight) that tends to remove contacts between node pairs with few contacts and removes early contacts seems to mitigate the epidemic spreading the best, with respect to the average prevalence, the peak prevalence and the time needed to reach the peak prevalence. This suggests that the removal of contacts along weak social ties in an early phase tends better suppress the epidemic spreading. Removing a large number of contacts from few node pairs is likely too costly to be effective. We demonstrate further that our finding, i.e., the 1/link weight strategy tends to outperform, still holds when uncertainty is introduced into original temporal networks via reshuffling the ordering of contacts and enlarging the temporal resolution, respectively.

Characterization of the pruned network resulted from the contact removal of a given strategy provides insights why some strategies outperform the others: an optimal strategy (1/link weight) leads to an aggregated pruned network with a large largest eigenvalue, a large modularity and a possibly small largest connected component size. A strategy tends to perform better when a similar number of contacts are removed from links. These findings are in line with our understanding that a network with a small largest connected component, a large modularity prohibits epidemic spreading. However, the large largest eigenvalue achieved by the optimal strategy seems to contradict our understanding that a static network with a large largest eigenvalue tends to facilitate SIS epidemic spreading with respect to its small epidemic threshold. We explain this seemingly inconsistency with respect to the difference between SIR and SIS models, between epidemic threshold and prevalence, and the complexity introduced by the temporal contacts that cannot be captured by the aggregated network.

A few limitations of our work should be noticed and could be explored in future work. First, we have confined ourselves to the SIR model with limited choice of parameters and a few real-world networks. SIR model is a simplified model of the epidemic spreading process, whereas real-world epidemic spreading can be more complicated. Hence, our conclusion regarding the effectiveness of the mitigation strategies cannot be generalized directly to real-world epidemic mitigation. It is essential to explore further generalized choice of more realistic epidemic spreading model. Second, the dependency of removal preference $p_{ij}(t)$ on time, i.e., *f*(*t*), that we have we chosen is one of the simplest forms. Other forms of time-dependent function *f*(*t*) could be further explored, especially those that are feasible for policy makers. The contact removal strategies proposed is based on the knowledge of the aggregated network over the observation window, the period when we intervene the spreading process. One challenging question is how to estimate or predict this aggregated network based on the observation of the aggregated network in the past. Beyond the aggregated network, contact removal strategies can also be based on temporal and topological properties of contacts.

## Data Availability

We have used publicly available data sets. More information of these data sets are provided in the corresponding references.

## Footnotes

1 In the simulation, the average number of contacts removed per link by strategy 1/link weight may differ slightly among the links. When the removal preference $p_{ij}>1$, we set removal probability $\tilde{p}_{ij}=1$ and iteratively re-normalize the removal probabilities of the other links to ensure that a fraction $\phi$ of contacts are removed.

2 Given a temporal network and a contact removal strategy, we have simulated per seed node 5 realizations of contact removal and a SIR spreading process on each resultant pruned network. The $\lambda _{1}(W^*)$ in the scatter plot is the average over the 5*N* realizations of the pruned network.
